# Comparative LCA Analysis of Selected Recycling Methods for Carbon Fibers and Socio-Economic Analysis

**DOI:** 10.3390/ma18112660

**Published:** 2025-06-05

**Authors:** Nikolina Poranek, Krzysztof Pikoń, Natalia Generowicz-Caba, Maciej Mańka, Joanna Kulczycka, Dimitrios Marinis, Ergina Farsari, Eleftherios Amanatides, Anna Lewandowska, Marcin Sajdak, Sebastian Werle, Szymon Sobek

**Affiliations:** 1 Department of Technologies and Installations for Waste Management, Faculty of Energy and Environmental Engineering, Silesian University of Technology, Konarskiego 18, 44-100 Gliwice, Poland; krzysztof.pikon@polsl.pl; 2 Mineral and Energy Economy Research Institute Polish Academy of Sciences, Wybickiego 7A Street, 31-261 Krakow, Poland; ngenerowicz@min-pan.krakow.pl (N.G.-C.); mmanka@min-pan.krakow.pl (M.M.); 3 Faculty of Management, AGH University of Science and Technology, Mickiewicza 30, 30-059 Krakow, Poland; kulczycka@min-pan.krakow.pl; 4 Department of Chemical Engineering, University of Patras, 26504 Patra, Greece; marinis@chemeng.upatras.gr (D.M.); efarsari@chemeng.upatras.gr (E.F.); lef@chemeng.upatras.gr (E.A.); 5 Institute of Management, Poznan University of Economics and Business, al. Niepodleglości 10, 61-875 Poznan, Poland; anna.lewandowska@ue.poznan.pl; 6 Department of Air Protection, Silesian University of Technology, Stanisława Konarskiego 22B, 44-100 Gliwice, Poland; marcin.sajdak@polsl.pl; 7 Department Thermal Technology, Silesian University of Technology, Stanisława Konarskiego 22, 44-100 Gliwice, Poland; 8 Department of Heating, Ventilation and Dust Removal Technology, Silesian University of Technology, Stanisława Konarskiego 20, 44-100 Gliwice, Poland; szymon.sobek@polsl.pl

**Keywords:** carbon fiber, environment, life cycle management, solvolysis, waste management

## Abstract

Carbon fiber is essential in many industries. Since primary production is highly energy-intensive, recycling technologies are being sought. A goal of the research was to develop at a laboratory scale a chemical recycling method aimed at recovering carbon fiber. Two variants of the method have been established and environmentally compared with a primary production version. Methods: The life cycle assessment methodology has been used to assess and quantify the environmental impacts. The cradle to gate analysis was performed with the functional unit defined as a production of 1 kg of carbon fiber. Results: The best environmental option turned out to be a developed chemical recycling technology named Scenario 1. It is a solvolysis performed using an ambient-pressure-operated batch reactor connected to a reflux condenser and an inert gas supply tank, using an ethylene glycol and potassium hydroxide solution. The worst case appeared to be the second variant of the chemical recycling, named Scenario 2 (plasma-enhanced nitric acid solvolysis). Conclusions: In Scenario 1, a production of the ethylene glycol was recognized as a key environmental driver, while in Scenarios 2 and 3 the energy-related impact was the most influential.

## 1. Introduction

The research and development of zero-carbon energy sources have become top priorities for countries worldwide since the beginning of the 21st century, largely due to the ongoing use of fossil fuels and significant greenhouse gas emissions [1]. According to the Ellen MacArthur Foundation report, the implementation of a circular economy (CE) could result in material savings of up to USD 630 billion annually by 2025 [2]. Transitioning to a CE could contribute to a reduction of global greenhouse gas emissions by 9.3 billion tons annually by 2050, which is equivalent to about a quarter of global annual emissions [3].

The production of carbon fiber components is experiencing significant growth, driven by increasing demand for lightweight and durable materials across various industries. In 2023, the global carbon fiber market was valued at approximately USD 7.1 billion and is projected to reach $23.2 billion by 2033, reflecting a compound annual growth rate (CAGR) of 12.6% [4,5,6].

This upward trend is particularly evident in sectors such as aerospace, automotive, and wind energy, where the emphasis is on reducing structural weight while maintaining high strength and energy efficiency. For instance, in the aerospace industry, carbon fiber’s superior strength-to-weight ratio makes it ideal for manufacturing aircraft components, contributing to reduced fuel consumption and emissions. Similarly, in the automotive sector, the use of carbon fiber enhances vehicle performance and fuel efficiency. In the wind energy industry, carbon fiber is utilized in the production of turbine blades, benefiting from its strength and lightweight properties [7].

Overall, the growing environmental awareness and pursuit of technological innovation are further propelling the expansion of the carbon fiber market. Carbon fiber finds widespread applications in the market. It is used, among other things, to manufacture a torque transmission shaft, which serves as a research material [8]. A torque transmission shaft is a mechanical component designed to transfer rotational force (torque) from one part of a system to another, commonly found in machinery, vehicles, and industrial equipment where power needs to be transmitted efficiently and reliably [9]. Its primary functions include transferring power from a source such as an engine or motor to another component like a gearbox or wheels, absorbing and distributing loads without excessive deformation or failure, and ensuring smooth rotation to prevent vibrations and mechanical issues. There are various types of torque transmission shafts, including solid shafts made of steel or alloy for high strength and durability, hollow shafts that reduce weight while maintaining strength, flexible shafts used in systems with misalignment, and splined shafts with grooves or teeth for precise torque transfer without slippage. These shafts must be made of strong, fatigue-resistant materials such as carbon steel, alloy steel, stainless steel, or composite materials for lightweight applications. Key design considerations include diameter and length to withstand torque and bending forces, surface finish to minimize friction and wear, and proper balancing and alignment to prevent vibrations and mechanical failures. Torque transmission shafts are widely used in automotive applications (as drive shafts, axles, and propeller shafts), in industrial machinery (like conveyor belts, pumps, and compressors), in aerospace engineering (where lightweight and strong materials are crucial), and in robotics for precision movement control. A well-designed torque transmission shaft ensures efficient power transfer, longevity, and reliability in mechanical systems [10].

Carbon fiber components are valued for their exceptional strength-to-weight ratio, corrosion resistance, and durability. They consist of 92–99% carbon atoms, and their carbon fiber-reinforced polymers (CFRPs) exhibit high tensile strength (up to 7 GPa) and a tensile modulus ranging from 230 to 600 GPa, making them significantly stronger yet lighter than steel [11,12]. These materials can reduce component weight by up to 70% while maintaining excellent fatigue resistance and thermal stability, withstanding temperatures up to 2000 °C in non-oxidizing environments. However, carbon fiber production is highly energy-intensive, consuming 20–30 times more energy per unit mass than steel production [12,13]. Additionally, recycling CFRPs remains a challenge due to their thermoset resin matrix—current methods such as pyrolysis, solvolysis, and mechanical grinding face efficiency and cost barriers. Due to their exceptional durability, these materials are difficult to recycle, especially carbon fibers embedded in resin, which complicates their recovery and reuse. Since the use of carbon fiber components is becoming increasingly desirable, the amount of waste generated will continue to grow in the market, making it crucial to find a recycling method that provides the greatest environmental relief [13,14]. Table 1 shows examples of different limitations in carbon fiber recycling.

CFRP (Carbon Fiber Reinforced Polymer) is a relatively new material on the market. In 2000, global CFRP production was approximately 10,000 tons per year. By 2010, this figure had increased to around 40,000 tons annually. Forecasts suggest that by 2025 production will exceed 200,000 tons per year [18,19,20].

The lifespan of CFRP products depends on the sector. For example, the estimated service life is 10–20 years in the automotive industry, 20–30 years in aviation, and 20–25 years in the wind energy sector. It is estimated that more than 172,500 tons of CFRP waste are generated [18,21].

Carbon fiber components are built from multi-layer laminates made of carbon fibers and a thermosetting polymer matrix. Adhesives or foams, used to partially fill certain parts, may also be involved in their assembly. Resin is a thermosetting material that undergoes a permanent chemical transformation, unlike thermoplastics, which presents a major challenge for recycling. Parts of boats, cars, and airplanes are made from these highly durable composite materials. Wind turbines account for only 5–10% of global composite material consumption. Therefore, research into the recycling of materials containing carbon fiber will also contribute to the development of recycling in other industries [22].

**Figure 1:**
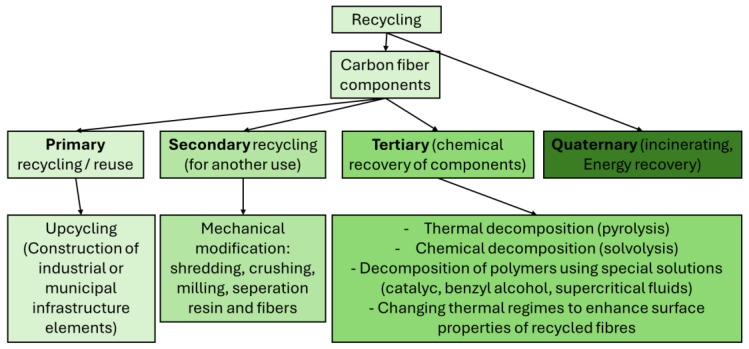
Possible recycling methods: Reuse, repurpose, decomposition, energy recovery [23].

The waste management hierarchy includes options ranked from least to most preferred: disposal → energy recovery → recycling → repurposing for other uses, reuse → reducing waste generation and prevention. Figure 1 presents a diagram of the available methods for processing wasted carbon fiber components [23].

As indicated in Figure 1, the wasted carbon fiber components may be reused (after preparing for re-use), recycled, or be a subject of energy recovery. A valuable option is a primary (closed loop) recycling where the wasted components are mechanically processed to obtain a material with the same function and with no losses in quality. Secondary (open loop) recycling is also a possible scenario. In this case, the waste is mechanically transformed into materials with different properties and functions. The carbon fiber components can be shredded and used as fillers, for instance, in concrete mixtures. One ton of carbon fiber component waste can reduce CO_2_ emissions by 110 kg and save 461 kg of raw materials compared to standard cement production. However, this approach wastes the structural properties of the material. Cut materials can also serve as fillers for vehicles or bicycles (reducing weight and lowering CO_2_ emissions) [24,25]. Tertiary recycling is another solution where the wasted materials are used as a feedstock in a process aimed at creating chemicals or fuels. An example of tertiary recycling is pyrolysis. This process involves heating the material to a temperature of 400–700 degrees Celsius in a low-oxygen environment. This breaks down the resin into simpler substances, allowing the fibers to be recovered and reused. Research shows that after heating the strength of the fibers can decrease by as much as 50%. Additionally, the pyrolysis process is energy-intensive and does not allow for resin recovery [26,27]. However, pyrolysis is being developed further. A Dutch team has developed a technology for extracting fibers from blades by briefly introducing oxygen into the process. Before oxygen is introduced, the resin and fibers are separated through oxygen-free pyrolysis. These recovered fibers can be used in automotive parts. Fibers recovered using the two-stage technology demonstrated 19% better tensile strength and 43% better fracture toughness compared to fibers recovered through single-step high-temperature pyrolysis [27,28].

Another tertiary recycling method is microwave processing. In this process, glass fibers can lose about 25% of their strength. An ideal recycling process should enable the recovery of both fibers and resin to produce new products. At the same time, the strength of the fibers should be comparable to that of virgin fibers. Chemical decomposition is another option for tertiary recycling. A review of the literature suggests that the solvolysis process may hold potential for recycling of the carbon fiber components. In the solvolysis process, the polymer matrix of the composite is swelled using volatile, high-boiling, low-molecular-weight solvents or solvent mixtures. Depending on the type of resin used in the composite, a catalyst may also be required. This catalyst, aided by the loosened composite matrix, can more effectively penetrate deep into the material and facilitate its degradation. The process occurs at an elevated temperature, restricted by the solvent’s boiling point, within a nitrogen-rich atmosphere at atmospheric pressure [29,30].

Chemical recycling has the potential to provide cleaner fibers while preserving more strength due to the lower temperatures involved compared to pyrolysis. Various solutions can be employed to break down polymers, such as catalytic processes, nitric acid, ammonia, glycol, and ethanol. In solvolysis processes, the phenomenon of the critical temperature can be utilized. This is the temperature above which the difference in density between the gaseous and liquid states of a substance disappears, making it impossible to liquefy the gas despite increasing pressure.

Solvolysis processes can also be combined with other techniques, such as microwaves or pyrolysis. Microwave-assisted chemical recycling processes can be used to recover glass fibers from composite waste, with the recovered fibers retaining 93–99% of the strength of virgin fibers. The recovered glass fibers demonstrated 99% tensile strength, 93% Young’s modulus, and 95% strain-to-failure compared to virgin fibers [28]. Different technologies consume varying amounts of materials, reagents, and energy needed for processing. This affects not only costs but also the environmental impact [28,29,30].

The aim of this study is to perform a life cycle assessment (LCA) comparing two recycling methods for carbon fiber-reinforced composites with the production of components made from virgin carbon fiber. The recovered carbon fibers originate from the recycling of a torque transmission shaft, as described in a later section of the article. The data for virgin carbon fiber were sourced from the Ecoinvent database (entry: Carbon fiber reinforced plastic, injection molded {GLO} | carbon fiber reinforced plastic, injection molded | Cut-off, U). The analysis was carried out using SimaPro software (PRé Sustainability, Stationsplein 121, Amersfoort, The Netherlands).

## 2. Materials and Methods

Environmental analyses are the foundation for a rational, evidence-based decision-making process. This is an important issue, closely linked to the economy. Proper decision-making processes determine vast financial flows directed towards investments. They also influence the mitigation of negative environmental impacts, which can be avoided or minimized in a much more effective way than by implementing many advanced technologies [29,30].

Environmental costs are difficult to estimate due to the lack of market pricing, and thus they are considered typical external costs that occur when one party’s profit or utility is impaired by the actions of another, without compensation. For this reason, purely economic approaches, which require the monetization of all effects, are often debated. A significant challenge in estimating environmental effects is the considerable time gap between the occurrence of an event and its environmental consequences. For example, the effects (and thus the environmental costs) of phenomena such as global climate change will be felt by many generations to come [31,32,33].

The life cycle assessment (LCA) is a sophisticated ecodesign tool and an element of environmental management systems [34,35]. The group of ISO 14040s standards include guidelines and requirements for performing LCA [36,37]. The LCA can be characterized by the following features: life cycle oriented, input–output related, environmentally multicriterial, impact assessment based, quantitative, scientifically grounded, standardized, and widely recognized. The LCA enables assessing and quantifying potential environmental impacts in the life cycle of goods and services. Additionally, it may be used to evaluate technologies and organizations. The analysis includes four phases: a goal and scope definition, a life cycle inventory (LCI), a life cycle impact assessment (LCIA), and an interpretation. More information about life cycle assessment methodology can be found in the literature [38,39].

### 2.1. Goal and Scope Definition

A main goal of the presented research was to develop in a laboratory scale a chemical recycling method aimed at recovering from wasted material a reclaimed carbon fiber. The method is based on the solvolysis and has been assumed as a potentially environmentally friendly alternative for a primary production. The wasted materials investigated for the different routes of the recycling were the torque transmission shafts (B&T Composites, Florina, Greece) cut to 20 cm length and 8 cm outer diameter comprising carbon fiber epoxy composite matrix. The samples were processes in the “as received” state, without any further cutting, milling, or other pretreatment methods.

Two variants of the recycling method have been assumed. In Scenario 1, a solvolysis process for the waste composite recycling was performed using a 0.5 and 2 L ambient pressure operated batch reactors connected to a reflux condenser and an inert gas supply tank (N2), using an ethylene glycol (EG) and potassium hydroxide (KOH) solution. The process was presented and discussed in detail in [27]. In Scenario 2, solvolysis was performed using plasma-enhanced nitric acid solvolysis of carbon fiber component composite scrap. This process synergistically combines the traditional nitric acid solvolysis with plasma aiming at the significant increase in the recovery rate of CFs. More details of the process can be found in [40].

The specific goals of the comparative LCA study are as follows:

- To evaluate the potential environmental impact and identify key drivers for the developed recycling processes (Scenario 1 and 2);
- To compare the recycling of the secondary carbon fiber with the primary production.

The potential audience of the results are all parties interested in ecodesign, carbon fiber production, and waste management.

The function of the recycling processes can be perceived from two perspectives: as disposing of waste (the end of life) and as generating secondary materials (the cradle). In our case study, there is a focus on the second function and the functional unit (FU) has been defined as a production of 1 kg of carbon fiber. The multifunctionality of recycling calls for the allocation procedure [41]. The developed recycling process combines two life cycles: the previous life cycle where the primary carbon fiber was incorporated in the torque transmission shafts and the subsequent life cycle where the reclaimed secondary carbon fiber will be used in similar or other applications. The carbon fiber included in the transmission shafts was based on a primary material, and for this reason, a virgin production was previously used for this purpose. This virgin material was present in the wasted shafts that were a subject of recycling. A heart of allocation is the following question, “how to allocate the environmental burdens related to the virgin production and to the recycling process between the previous and subsequent life cycles?”. There can be different answers, as many allocation approaches exist. In our case study, the cut-off approach has been used. This means that 100% virgin production and 0% recycling has been allocated to the previous life cycle (transmission shafts), and 0% virgin production and 100% recycling has been allocated to the subsequent life cycle (where the reclaimed carbon fiber will be used). No credits (avoided burdens) have been accounted for the recycling.

**Figure 2:**
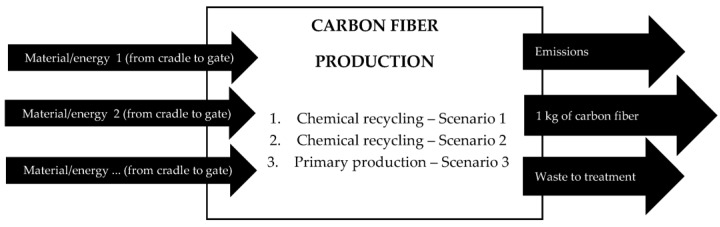
The system boundaries of LCA.

**Figure 3:**
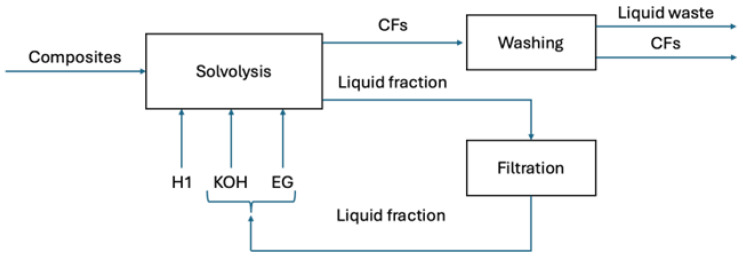
Schematic diagram of the solvolysis process for Scenario 1.

**Figure 4:**
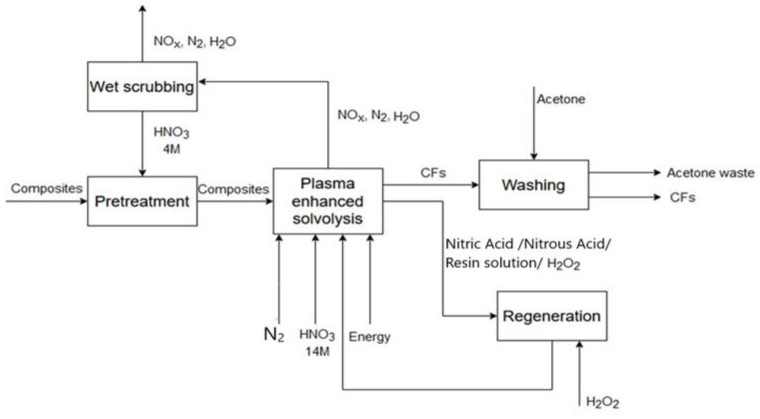
Schematic diagram of the solvolysis process for Scenario 2.

The LCA analysis has been scoped from the cradle to gate. Figure 2 presents general system boundaries. More detailed information is presented in process diagrams (Figure 3 and Figure 4). A transport of the wasted material to the laboratory has been included while a quality assessment between the primary and the reclaimed carbon fibers has been excluded from the analysis.

### 2.2. Life Cycle Inventory (LCI)

In the presented case study, two data sources have been used. The research presented in this study concerns torque transmission shafts, which were used as the representative waste material for evaluating various recycling methods. For both variants of recycling process (Scenarios 1 and 2) activity data have been modeled with primary information based on stoichiometric calculations and reactions made in the laboratory. The data can be recognized as good quality in terms of temporal, geographical, and technological scope [42]. It means that they are consistent with the goal of the study. A main limitation lies in their reliability and completeness, as the data have been calculated based on activities made in a laboratory, not on a commercial scale. It makes it probable that the activity data for Scenarios 1 and 2 could be overestimated. The activity data for the primary production (Scenario 3) have been taken from the ecoinvent dataset carbon fiber reinforced plastic, injection molded (GLO). All background processes have been modeled with the ecoinvent v3.10 secondary data. Schematic process diagrams and inventory results for Scenarios 1 and 2 are presented in Figure 3 and Figure 4 and Table 2 and Table 3.

### 2.3. Life Cycle Impact Assessment (LCIA)

The life cycle impact assessment calculations have been made with the Environmental Footprint EF 3.1 method (adapted) v3.10, available in the SimaPro software. The LCIA method has been developed by the European Commission as an element of Environmental Footprints methodology [43]. With this method, the environmental impact is to be assessed within 16 impact categories reflecting various environmental problems: Acidification, Climate change, Ecotoxicity-freshwater, Particulate matter, Eutrophication-marine, Eutrophication-freshwater, Eutrophication-terrestrial, Human toxicity-cancer, Human toxicity-non-cancer, Ionizing radiation, Land use, Ozone depletion, Photochemical ozone formation, Resource use-fossils, Resource use-minerals and metals and Water use. LCIA results are presented in Section 3.

## 3. Results

**Figure 5:**
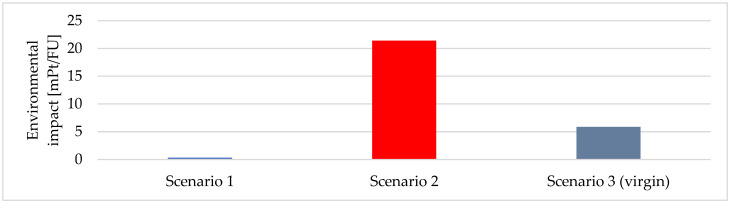
The potential environmental impact (single score) for the three scenarios [mPt/FU].

Two sorts of the results are presented in Section 3: characterized (where each impact category has its own unit) and weighted (all impact categories have the same unit-milipoints). In both cases the same interpretation rule works. The higher the value of the indicator, the higher the negative environmental impact is. Table 4 and Figure 5 show the weighted results for production of 1 kg of carbon fiber with three processes: the chemical recycling (Scenario 1 and Scenario 2) and primary production (Scenario 3). The cumulated impact (single sore) is 0.33 mPt for Scenario 1, 21.44 mPt for Scenario 2, and 5.85 mPt for Scenario 3 (the primary production). The results clearly show that Scenario 1 has the lowest environmental impact while Scenario 2 is the worst option. In Table 4, the cells marked in gray indicate the most relevant impact categories (the categories with the highest contribution).

**Figure 6:**
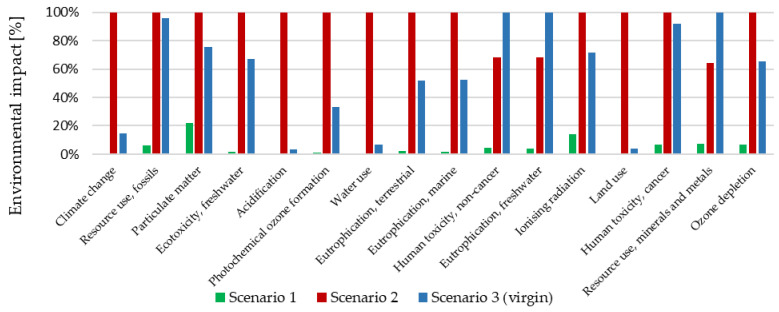
The environmental impact (characterized) for three Scenarios under analysis. Source: SimaPro software.

Table 5 and Figure 6 present the environmental impact as the characterized results. It can be observed that Scenario 1 is the best option for all impact categories.

**Figure 7:**
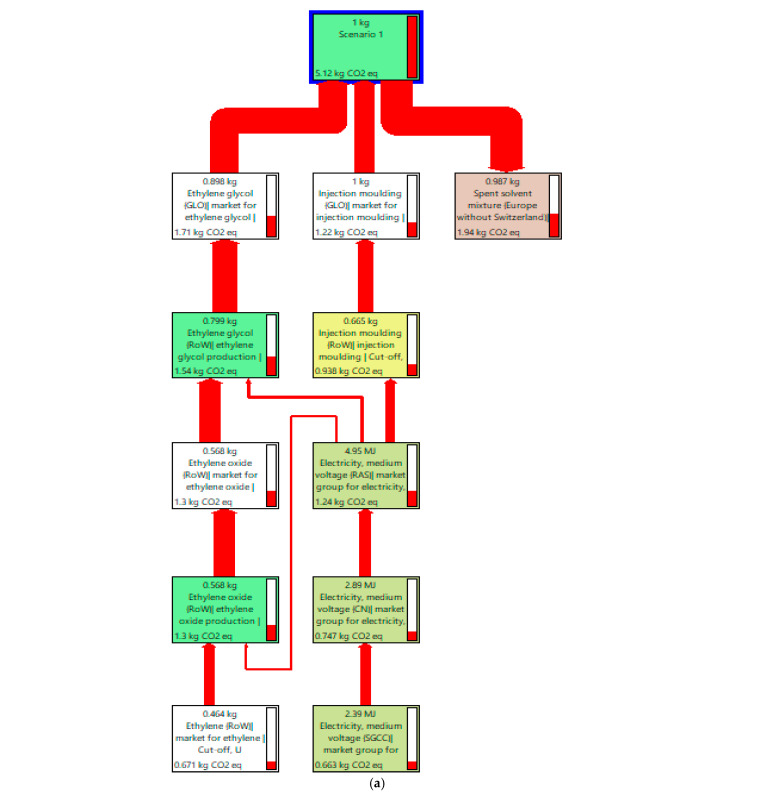

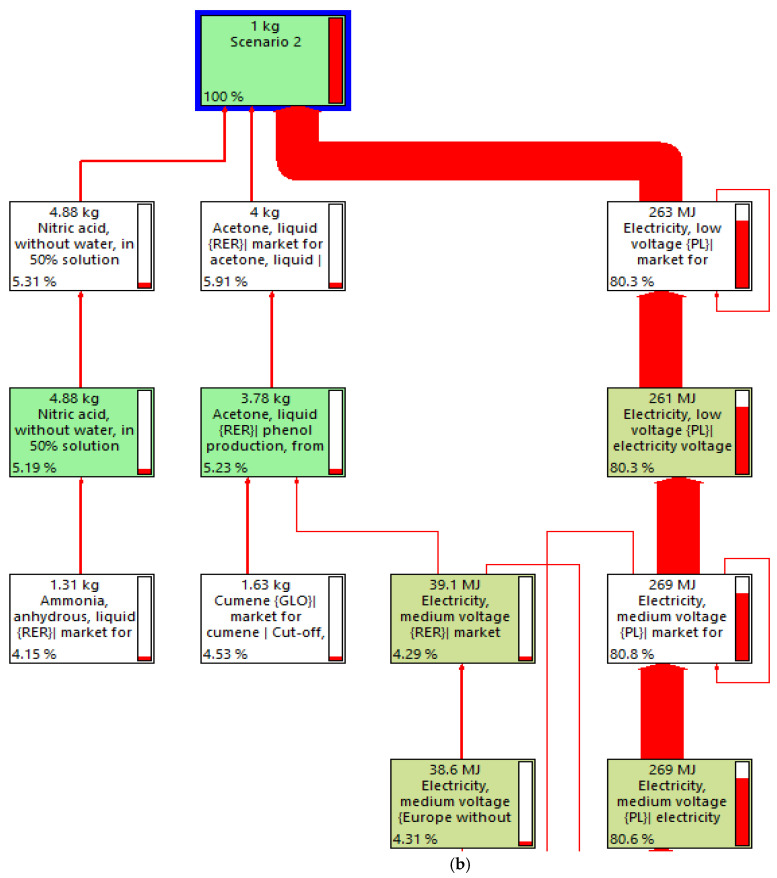

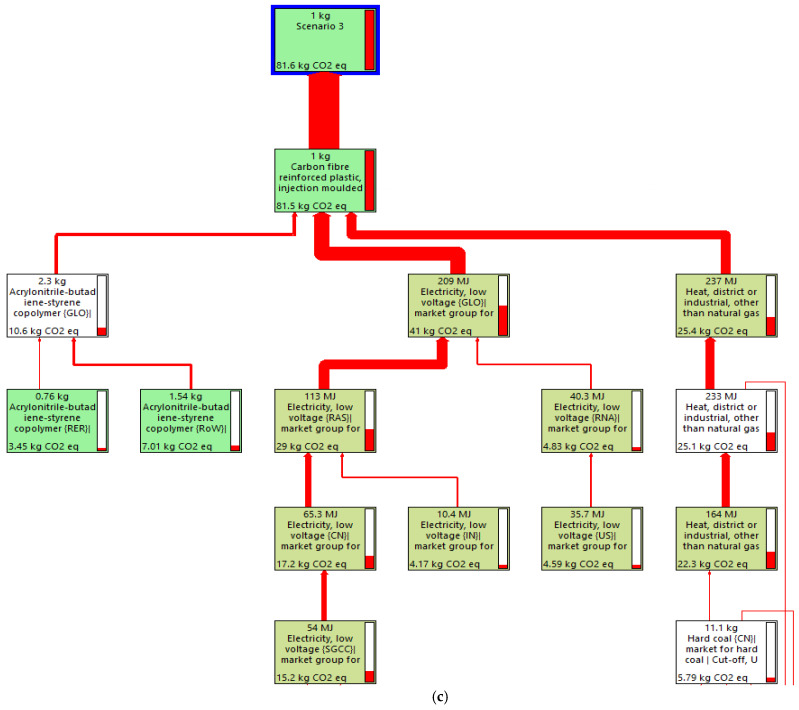
Flow diagrams reflecting the processes contributing mostly to the Climate Change (kg CO_2_ eq/FU) (**a**) Scenario 1; (**b**) Scenario 2; (**c**) Scenario 3. Source: SimaPro software.

Based on the data from Table 4 and Figure 6, the following key conclusions can be drawn. Scenario 1 clearly has the lowest environmental impact across all analyzed impact categories, including CO_2_ emissions (5.12 kg CO_2_ eq), acidification (0.0142 mol H^+^ eq), and fossil resource use (68.6 MJ). This makes it the most environmentally friendly option among the three considered. Scenario 2 generates the highest environmental burdens in most categories, particularly in terms of climate change (85.4 kg CO_2_ eq), eutrophication (freshwater, marine, and terrestrial), and land use (150 Pt). Scenario 3 ranks in the middle, falling between Scenarios 1 and 2. Although it has a lower impact than Scenario 2 in most categories, it is still significantly more environmentally burdensome than Scenario 1, especially in terms of CO_2_ emissions (81.6 kg CO_2_ eq) and fossil fuel consumption (976 MJ). Figure 7 shows flow diagrams of the three scenarios in the impact category Climate Change.

For Scenario 1, the most influential factor is Ethylene glycol, which accounts for over 33% of the result. In Scenario 2, electricity has the greatest impact on the results, representing over 80% of the total impact. In Scenario 3, electricity also has the largest environmental impact, but it accounts for 50% of the result (209 MJ).

## 4. Socio-Economic Analysis

Recycling significantly influences the economy through job creation, increased competitiveness, cost savings, and overall improvements in societal well-being. It generates employment across various sectors, including waste collection, material processing, and manufacturing of new products from recycled materials, with industries such as wind energy and automotive manufacturing directly benefiting from secondary raw materials. This process not only reduces dependence on imported resources but also lowers production costs, enhancing the economy’s resilience. Furthermore, innovation in recycling technologies fosters the development of new industrial sectors, driving long-term growth and sustainability.

Despite the costs of establishing waste collection systems and processing facilities, recycling brings financial benefits by reducing landfill usage and lowering expenses tied to raw material extraction. Beyond economics, recycling boosts environmental awareness through education and public campaigns, encouraging more sustainable consumption habits. Health benefits also arise from decreased pollution, leading to better air, water, and soil quality, and lowering pollution-related disease rates. In terms of quality of life, recycling supports cleaner surroundings, reduces illegal dumping, and promotes the circular economy by increasing access to valuable secondary materials.

However, challenges persist, such as high infrastructure costs, uneven access to recycling facilities, and low public motivation due to insufficient information systems. Addressing these barriers is essential to maximizing the socio-economic potential of recycling and building a more sustainable, circular economy. Table 6 shows the possible impact of recycling on the economy and the community.

### Carbon Fiber Market

The carbon fiber market has been growing dynamically in recent years, driven mainly by demand in the aerospace, automotive, and wind energy sectors. In 2019, the market was valued at USD 4.7 billion, with forecasts suggesting it could grow to USD 13.3 billion by 2029, representing a compound annual growth rate (CAGR) of 11% [44].

**Figure 8:**
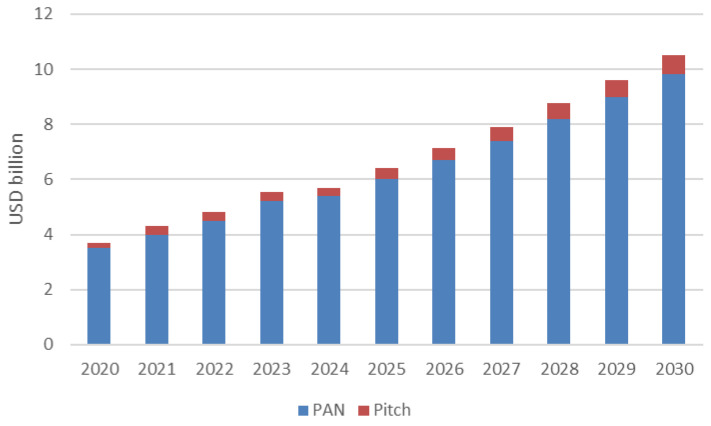
Carbon fiber market.

Figure 8 shows the size of the global carbon fiber market from 2020 to 2030, broken down by raw material: PAN (polyacrylonitrile) and pitch, with values in USD billion. The data indicate steady market growth, with the total size increasing from USD 3.7 billion in 2020 to USD 10.5 billion in 2030. PAN remains the dominant raw material, consistently accounting for the majority of the market share, while pitch contributes a smaller but gradually growing portion. The market experiences consistent year-over-year growth, with a notable compound annual growth rate (CAGR) of 10.9% from 2025 to 2030. This expansion reflects rising demand for carbon fiber across industries such as aerospace, automotive, wind energy, and sporting goods.

In terms of value, the global carbon fiber market is expected to reach approximately USD 8.5 billion by 2030 [45]. Earlier estimates from 2019 suggested the market could hit USD 8 billion by 2026, with a CAGR of 10.8%.

Geographically, the market spans key regions, including Europe, Asia-Pacific, North America, the Middle East and Africa, and Latin America. While the demand is rising, the market faces challenges, including the high cost of carbon fiber and the lack of standardized manufacturing technologies, which may slow down wider adoption.

The competitive landscape highlights Toray Industries Inc. as a leading player, with various other companies positioned across different levels of market share and product footprint. The ecosystem involves raw material providers like Minglang and Lanhai, carbon fiber manufacturers such as Toray and SGL Carbon, and end users including major global brands like Teijin, Mercedes, and Audi.

Overall, the data suggests that while the carbon fiber market holds immense growth potential, its expansion depends on addressing cost barriers and streamlining production processes to meet rising global demand.

The key drivers of this growth are the push to reduce vehicle and structure weight to improve energy efficiency and lower emissions. Carbon fibers, thanks to their lightweight and high-strength properties, are widely used in aerospace, automotive manufacturing, wind energy, and sports and recreational equipment production [4,5,6,7].

## 5. Discussion

This study was conducted to compare two carbon fiber recycling methods with virgin production using environmental gate-to-gate life cycle assessment (LCA) methodologies. The results reveal that not all recycling processes have a lower environmental impact than virgin carbon fiber production. However, the research identified that the most environmentally favorable method is a developed chemical recycling technology, referred to as Scenario 1. This process involves solvolysis performed in ambient-pressure batch reactors, equipped with a reflux condenser and an inert gas supply, using a solution of ethylene glycol and potassium hydroxide.

Scenario 2 is still under development, and therefore it is possible that it could also demonstrate a lower environmental impact than virgin carbon fiber production. Sensitivity analysis plays an important role in all scenarios, helping to identify and reduce environmental hotspots. In Scenario 1, ethylene glycol emerges as the most impactful factor, contributing over 33% to the total environmental burden. In Scenario 2, electricity has the highest impact, accounting for over 80% of the total footprint. Similarly, in Scenario 3, electricity remains the most significant factor, representing 50% of the impact (equivalent to 209 MJ).

If energy is the hotspot, it can potentially be replaced with renewable or waste-derived energy sources. If the hotspot is a chemical substance, exploring potential substitutes could be a viable solution. These considerations could serve as the foundation for future research directions.

The article also discusses other recycling processes, as carbon fiber recycling is increasingly relevant due to growing market demand. The expanding use of carbon fiber across industries—including aerospace, automotive, wind energy, and sports equipment—is expected to substantially increase the volume of waste generated both during production and at the end of product life. Therefore, there is a pressing need to develop and deploy innovative, sustainable strategies to manage this waste stream.

The average annual growth in demand for carbon fiber-reinforced composites (CFRCs) is estimated at approximately 11%.

One key strategy is to explore and refine carbon fiber recycling methods. However, not all recycling processes are inherently environmentally beneficial, which is why conducting a life cycle assessment (LCA) is crucial. LCA enables a comprehensive evaluation of environmental impacts across the entire process—including resource use, energy consumption, emissions, and waste generation. Through such analysis, it becomes possible to assess whether a recycling method truly offers environmental advantages compared to traditional disposal or the use of virgin materials.

A socio-economic analysis highlights a gap in the market that could be filled if the carbon fiber recycling industry expands. Potential benefits include the creation of new jobs, increased market competitiveness, and reduced pollution levels. Given that the global carbon fiber market is projected to reach approximately USD 8.5 billion by 2025, future studies should explore the exact number of new jobs that could be generated and assess the health impact on workers from implementing cleaner recycling technologies.

It is especially important to evaluate a specific recycling process in direct comparison with virgin carbon fiber production. The global production of virgin carbon fiber is dominated by several major manufacturers known for their advanced materials and extensive production capacities. Leading companies in this sector include Toray Industries (Tokyo, Japan), one of the largest and most established producers [46]; Zoltek (a subsidiary of Toray, St. Louis, MI, USA), specializing in industrial-grade carbon fibers; Mitsubishi Chemical (Tokyo, Japan), known for high-performance fibers used in aerospace and automotive industries [47]; Hexcel Corporation (Stamford, CT, USA), a key supplier for aerospace composites [48]; and SGL Carbon (Wiesbaden, Germany), which serves diverse markets including automotive and wind energy [49]. These producers contribute significantly to the global supply chain, driving innovation and scaling production to meet growing demand. However, for the purposes of life cycle assessment and environmental impact calculations in this study, secondary data from the Ecoinvent database was employed to ensure consistency and comparability. This is particularly relevant in light of the new ISO 14075:2024 standard published at the end of 2024, which reinforces the growing trend in economic thinking—not solely focused on profit, but increasingly on environmental sustainability and human well-being, including worker health.

Nevertheless, due to the high cost of virgin carbon fiber, there is a growing need to develop new and more cost-effective solutions. Recycling carbon fiber composites plays a crucial role in addressing this challenge. Although the concept of recycling is not entirely new, several companies have already established industrial-scale recycling operations—for example, ELG Carbon Fibre Ltd., Coseley, UK [50]. Their efforts not only demonstrate the feasibility of recycling but also highlight the ongoing need for improved, economically viable recycling technologies to meet increasing demand sustainably.

While the composites industry must thoroughly evaluate the economic and environmental aspects of carbon fiber composite recycling, it is equally important to acknowledge the critical role of the resin component. The type of resin used significantly influences the choice and effectiveness of the recycling method. Although this factor is not addressed in the current article, future research may place strong emphasis on resin analysis, as it represents a key element in developing efficient and sustainable recycling strategies.

In conclusion, while carbon fiber recycling is a promising path forward, only those solutions that demonstrate environmental benefits or are cleaner and safer for human health (as validated through tools like LCA) should be prioritized for large-scale implementation.

## 6. Conclusions

The recycling of carbon fiber-based components aligns with the principles of a circular economy and sustainable development. One method for recovering carbon fibers from components is solvolysis. However, research indicates that not all recycling processes necessarily benefit the environment—in some cases, producing material from virgin resources may have a lower environmental impact than recycling. Nevertheless, proper process optimization and hotspot identification can improve LCA results. Studies confirm the importance of environmental analyses to select processes that truly reduce environmental impact.

The results show that, at the current stage of research, among the two analyzed recycling (solvolysis) processes, Scenario 2 has the highest negative environmental impact compared to carbon fiber production from virgin materials (Scenario 3). Additionally, Scenario 2 has a specific hotspot (electricity consumption by water bathes used to generate heat), which could be optimized, for example, by utilizing waste heat.

Scenario 2 is still in the development stage, and its potential is continuously improving, offering opportunities for better performance and environmental outcomes through ongoing optimization and waste minimization. On the other hand, Scenario 1 provides environmental benefits compared to virgin fiber production.

The presented research constitutes a preliminary approach to the topic of chemical recycling of composite waste and demonstrates the potential of the studied process for their disposal and the recovery of valuable oligomers and fibers (in this example, carbon fibers). Our further research will focus on increasing the number of cycles in which the liquid solvolysis products are recirculated into the process, aiming to concentrate the oligomers released from the resin matrix. In the next stage, we will develop a method for purifying these oligomers and using them for the synthesis of new resin. The results have been presented at TRL 1–3, and future scale-up will enable us to address challenges related to energy recovery.

## Figures and Tables

**Table 1:** Examples of limitations of carbon fiber recycling for selected technologies.

Recycling Method	Description	Key Limitations	Source
Mechanical Recycling	Grinding/shredding CFRP into small particles for fillers or low-grade reuse	Fibers are short and damaged; Loss of mechanical properties; Low-value recycled product	[15]
Thermal Recycling	Pyrolysis or fluidized bed to burn off resin and recover fibers	High energy demand; Potential fiber surface degradation; Emission of VOCs without control	[16]
Chemical Recycling (Solvolysis)	Use of solvents under heat/pressure to dissolve matrix and recover clean fibers	Complex solvent handling; High process cost and time; Challenges in scaling up and purifying recovered oligomers	[17]

**Table 2:** Inventory results for recycling process-Scenario 1 (per functional unit).

Name of Input/Output	Amount	Unit
**Inputs**		
Wasted carbon fiber component (B&T Composites)	1.44	kg
Transport of wasted carbon fiber component, lorry 16–32, Euro 5 (100 km)	1.44 × 10^2^	kgkm
Solvent (closed looped circulation)	5.74	kg
Ethylene glycol	8.97 × 10^−1^	kg
Potassium Hydroxide	8.97 × 10^−2^	kg
Electricity (low voltage, at grid, Poland)	2.16	kWh
**Outputs**		
Reclaimed carbon fiber	1.00	kg
Solvent (closed looped circulation)	5.74	kg
Liquid waste	9.87 × 10^−1^	kg

**Table 3:** Inventory results for recycling process-Scenario 2 (per functional unit).

Name of Input/Output	Amount	Unit
**Inputs**		
Wasted carbon fiber component (B&T Composites)	1.67	kg
Transport of wasted carbon fiber component, lorry 16–32, Euro 5 (100 km)	1.67 × 10^2^	kgkm
Nitric Acid 65%/H_2_O_2_ 30% (closed looped circulation)	2.77 × 10^1^	kg
Nitrogen gas	1.63 × 10^1^	kg
Acetone	4.00	kg
Electricity (low voltage, at grid, Poland)	7.17 × 10^1^	kWh
**Outputs**		
Reclaimed carbon fiber	1.00	kg
NO_X_ gasses that enter the wet scrubber	6.83	kg
NO_X_ gasses that escape the wet scrubber	5.00	kg
Wet scrubbing solution (HNO_3_)	1.83	kg
Nitric Acid/Nitrous Acid/Resin solution/H_2_O_2_ (closed looped circulation)	2.08 × 10^1^	kg
Nitrogen gas	1.63 × 10^1^	kg
Acetone/resin residuals	4.00	kg

**Table 4:** The environmental impact per functional unit-weighted results for Scenarios 1–3.

Impact Category	Scenario 1		Scenario 2		Scenario 3 (Virgin)	
	mPt	%	mPt	%	mPt	%
**Single Score**	**3.31 × 10^−1^**	**100%**	**2.14× 10^1^**	**100%**	**5.85**	**100%**
Climate change	1.43 × 10^−1^	43%	2.38	11%	2.27	39%
Resource use, fossils	8.77 × 10^−2^	27%	1.36	6%	1.25	21%
Particulate matter	2.09 × 10^−2^	6%	1.38	6%	9.24 × 10^−1^	16%
Ecotoxicity, freshwater	1.79 × 10^−2^	5%	8.24 × 10^−2^	0.4%	6.21 × 10^−2^	1.1%
Acidification	1.58 × 10^−2^	5%	3.41	16%	5.02 × 10^−1^	9%
Photochemical ozone formation	1.58 × 10^−2^	5%	6.85	32%	2.71 × 10^−1^	5%
Water use	1.07 × 10^−2^	3%	1.60 × 10^−1^	0.7%	1.04 × 10^−1^	2%
Eutrophication, terrestrial	6.50 × 10^−3^	2%	2.38	11%	1.55 × 10^−1^	3%
Eutrophication, marine	4.20 × 10^−3^	1.3%	3.04	14%	1.03 × 10^−1^	2%
Human toxicity, non-cancer	2.70 × 10^−3^	0.8%	1.45 × 10^−1^	0.7%	7.58 × 10^−2^	1.3%
Eutrophication, freshwater	2.20 × 10^−3^	0.7%	1.67 × 10^−1^	0.8%	5.53 × 10^−2^	0.9%
Ionizing radiation	1.30 × 10^−3^	0.4%	1.96 × 10^−2^	0.1%	2.86 × 10^−2^	0.5%
Land use	9.00 × 10^−4^	0.3%	1.45 × 10^−2^	0.1%	2.13 × 10^−2^	0.4%
Human toxicity, cancer	8.00 × 10^−4^	0.2%	3.63 × 10^−2^	0.2%	1.89 × 10^−2^	0.3%
Resource use, minerals and metals	4.00 × 10^−4^	0.1%	3.60 × 10^−3^	0.02%	5.60 × 10^−3^	0.1%
Ozone depletion	1.00 × 10^−4^	0.03%	7.00 × 10^−4^	0.00%	5.00 × 10^−4^	0.0%

**Table 5:** The environmental impact per functional unit-characterized results for Scenarios 1–3.

Impact Category	Unit	Scenario 1	Scenario 2	Scenario 3 (Virgin)
Acidification	mol H^+^ eq	1.42 × 10^−2^	3.06	4.50 × 10^−1^
Climate change	kg CO_2_ eq	5.12	8.54 × 10^1^	8.16 × 10^1^
Ecotoxicity, freshwater	CTUe	5.30 × 10^1^	2.43 × 10^2^	1.83 × 10^2^
Particulate matter	disease inc.	1.39 × 10^−7^	9.17 × 10^−6^	6.14 × 10^−6^
Eutrophication, marine	kg N eq	2.80 × 10^−3^	2.01	6.77 × 10^−2^
Eutrophication, freshwater	kg P eq	1.00 × 10^−4^	9.60 × 10^−3^	3.20 × 10^−3^
Eutrophication, terrestrial	mol N eq	3.08 × 10^−2^	1.14 × 10^1^	7.40 × 10^−1^
Human toxicity, cancer	CTUh	6.48 × 10^−10^	2.94 × 10^−8^	1.53 × 10^−8^
Human toxicity, non-cancer	CTUh	1.89 × 10^−8^	1.01 × 10^−6^	5.30 × 10^−7^
Ionizing radiation	kBq U-235 eq	1.13 × 10^−1^	1.65	2.41
Land use	Pt	9.19	1.50 × 10^2^	2.20 × 10^2^
Ozone depletion	kg CFC11 eq	8.30 × 10^−8^	5.81 × 10^−7^	4.15 × 10^−7^
Photochemical ozone formation	kg NMVOC eq	1.35 × 10^−2^	5.86	2.32 × 10^−1^
Resource use, fossils	MJ	6.86 × 10^1^	1.06 × 10^3^	9.76 × 10^2^
Resource use, minerals and metals	kg Sb eq	3.37 × 10^−7^	3.03 × 10^−6^	4.72 × 10^−6^
Water use	m^3^ depriv.	1.44	2.15 × 10^1^	1.40 × 10^1^

**Table 6:** Possible impact of recycling on the economy and the community.

Category	Impact/Description
Job creation	Recycling generates employment in sectors such as collection, processing, and the production of new products from secondary raw materials.
Increase in economic competitiveness	The use of secondary raw materials reduces production costs and makes the economy less dependent on the import of primary raw materials.
	Innovations in recycling technology drive the development of new industrial sectors.
Costs and savings related to recycling	Costs of selective waste collection systems and processing technologies.
	Savings resulting from a reduced need for waste landfilling and lower costs of extracting natural resources.
Social environmental awareness	Environmental education and changing consumption habits.
	Social campaigns and local government initiatives promoting waste segregation.
Health benefits	Reduced environmental pollution improves air, water, and soil quality.
	Lower emissions of harmful substances decrease the incidence of pollution-related diseases.
Impact on quality of life	Recycling promotes a cleaner environment and reduces the number of illegal dumpsites.
	Increased availability of secondary raw materials supports the development of a circular economy.
Environmental awareness	Increasing environmental awareness influences the formation of more sustainable consumption habits, promotes waste segregation, and encourages responsible resource management. Environmental education and social campaigns inspire people to take everyday actions for environmental protection, such as reducing plastic use, reusing products, or choosing local and eco-friendly goods. Greater awareness makes society more likely to demand systemic change, support pro-environmental initiatives, and pressure companies and institutions to adopt more sustainable strategies. In the long run, this leads to improved quality of life, reduced pollution, and healthier ecosystems, benefiting both people and the planet.

## Data Availability

The original contributions presented in this study are included in the article. Further inquiries can be directed to the corresponding author.
